# Patient-reported outcomes to assess the efficacy of extended-release guaifenesin for the treatment of acute respiratory tract infection symptoms

**DOI:** 10.1186/1465-9921-13-118

**Published:** 2012-12-27

**Authors:** Helmut Albrecht, Margaret Vernon, Gail Solomon

**Affiliations:** 1 H2A Associates, LLC, 3350 SW 27th Ave, Ste 1803, Miami, FL, 33133, USA; 2 United BioSource, 7101 Wisconsin Ave, Ste 600, Bethesda, MD, 20814, USA; 3 Reckitt Benckiser Inc., 399 Interpace Parkway, Parsippany, NJ, 07054-0225, USA

**Keywords:** Expectorant, Extended-release guaifenesin, Clinical methods development, Patient-reported outcomes, Symptom improvement, Validation, Psychometric, Content validity, Upper respiratory tract infections, Thickened mucus

## Abstract

**Background:**

Guaifenesin is a component of medicines used to improve symptoms associated with upper respiratory tract infections. Patient-reported outcome instruments are valuable for evaluating symptom improvements; however, a validated tool to assess efficacy of mucoactive drugs does not exist. We compared the efficacy of extended-release guaifenesin with placebo for treatment of symptoms of upper respiratory tract infection using subjective efficacy assessments in a pilot study and confirmed precision of assessments in a validation study.

**Methods:**

The pilot study was a randomized, double-blind study where patients were dosed with either 1200 mg extended-release guaifenesin (n = 188) or placebo (n = 190), every 12 hours for 7 days. Efficacy was assessed using subjective measures including the Daily Cough and Phlegm Diary, the Spontaneous Symptom Severity Assessment and the Wisconsin Upper Respiratory Symptom Survey. End-of-study assessments were completed by patients and investigator. The validation study consisted of two phases. In Phase I, subjects completed interviews to gather evidence to support the content validity of the Daily Cough and Phlegm Diary, the Spontaneous Symptom Severity Assessment and Patient’s End-of-Treatment Assessment. Phase II examined the psychometric properties of assessments evaluated in Phase I of the validation study using data from the pilot study.

**Results:**

Subjective measures of efficacy at Day 4 showed the most prominent difference between treatment groups, in favor of guaifenesin. The 8-symptom related questions (SUM8) in the Daily Cough and Phlegm Diary, analyzed as a composite score appeared to be the strongest candidate endpoint for further evaluation. Results from the interviews in Phase I supported the content of the assessments which were validated during Phase II. Treatments were well tolerated.

**Conclusions:**

Results from the clinical pilot and validation studies showed that the SUM8 diary scores were robust and reliable for use as efficacy endpoints in studies of mucoactive drugs.

**Trial registration:**

The study was registered with clinicaltrials.gov (NCT01046136).

## Background

Expectorants, such as guaifenesin, are a component of many cough and cold medicines that are used to improve mucociliary clearance and relieve chest congestion associated with acute upper respiratory tract infections (URTIs) [1-4].

Objective measures and methods to assess the treatment effect of mucoactive products have been problematic and have shown inconsistent outcomes [5,6]. For evaluating any improvement in symptoms of an URTI, patient-reported outcome (PRO) instruments are a valuable method. However, during the clinical development of an extended-release (ER), bilayer formulation of guaifenesin (Mucinex, Reckitt Benckiser, Parsippany, NJ, USA), it became apparent that a universally accepted, validated PRO tool to assess the efficacy of mucoactive drugs does not exist.

The heterogeneous nature of URTIs and the daily changes that occur during the natural resolution of infections makes assessments of products to treat them challenging [7]. There is also the placebo effect associated with cough studies, which has been well documented [8,9]. The symptoms of URTIs are also highly subjective and PROs currently lack the precision to differentiate minimally important changes due to treatment from those occurring due to natural resolution of symptoms.

It is important from a regulatory perspective that any primary efficacy endpoint should be clinically relevant and appropriately validated. The US Food and Drug Administration and European Medicines Agency guidance for industry highlights the importance of using validated PRO measures when investigating new drugs [10,11].

In this manuscript we describe the results of a clinical pilot study where ER guaifenesin was compared with placebo for the treatment of symptoms of acute URTIs. We also report results from a validation study which consisted of two phases, used to confirm the precision of PROs used in the pilot study. The overarching objective of this pilot and validation research was to identify and subsequently verify suitable clinical instruments for measuring mucoactive treatment outcomes in future studies of patients with symptoms of acute URTIs.

## Methods

### Clinical pilot study

#### Study design and objectives

The clinical pilot study was a multicenter, randomized, parallel group, double-blind, placebo-controlled study to investigate the effects of 1200 mg ER guaifenesin (Mucinex), dosed every 12 hours for 7 days, on symptoms (using subjective measures) and sputum properties (using objective measures) in subjects with an acute URTI. The secondary objective was to determine the safety and tolerability of ER guaifenesin in this patient population.

Another key objective was to explore clinical endpoints and methods for potential follow-up studies. Exploratory analyses were conducted on composite subsets of questions chosen from among the 11 questions in the Daily Cough and Phlegm Diary (DCPD); the objective was to search for more sensitive efficacy endpoints and/or criteria to help identify a more refined patient population to better discriminate between active and placebo treatments in acute URTI patient studies. The 11-item DCPD consisted of an 8-question diary symptom subscale and 3 social function questions.

The pilot study protocol was submitted for independent ethical review, and approval was obtained in writing from the institutional review board (IRB), Quorum Review Inc. (Seattle, WA, USA). The study was conducted in accordance with the principles of the harmonized tripartite guideline E6 (R1) of the International Conference on Harmonisation, Good Clinical Practice, the Declaration of Helsinki, Title 21 of the United States Code of Federal Regulations, and applicable national, state, and local laws or regulations. All participants (or parents or legally authorized representatives) were required to provide written informed consent before starting the study.

#### Subject selection

Male and female volunteers aged ≥12 years were recruited from 12 sites in the USA if they had symptoms of an acute URTI diagnosed by the investigator within 5 days of onset. Study investigators were family physicians and specialists in asthma/allergy, otolaryngology and emergency medicine. The inclusion criteria required volunteers to have symptoms of moderate or greater severity for two of three symptoms of cough, thickened mucus or chest congestion as measured by the Spontaneous Symptom Severity Assessment (SSSA) score; have developed productive cough within 72 hours prior to dosing on Day 1, and be able to expectorate sputum. Volunteers were included if they met the above criteria, were otherwise healthy as determined by medical history and physical examination (including vital signs), and if the investigator felt they would be compliant and complete the study.

Volunteers were excluded from the study if they had chronic, recurring respiratory signs and symptoms due to allergic rhinitis or chronic bronchitis, asthma or wheezing; any significant disease of the heart, kidney, liver, lung, uncontrolled hypertension, or diabetes mellitus, cystic fibrosis or thyroid disorder; or any other disease which may have interfered with study outcomes or caused undue risk to the patient. Volunteers who had febrile illness (>101°F) within 7 days or received 2009 H1N1 influenza vaccine within 2 weeks of Day 1 were excluded. Pregnant or lactating women were excluded and females of childbearing age must have been using birth control for at least 3 months prior to the study; they were also required to provide a negative urine pregnancy test on Day 1.

Volunteers were not permitted to participate if they had taken part in another clinical investigation within 30 days before the baseline visit; had known hypersensitivity or allergy to guaifenesin or any product ingredients; were being treated with intranasal medications or systemic antihistamines, bronchodilators or decongestants; had used any over-the-counter (OTC) cough, cold, or allergy medication within 24 hours prior to Day 1 or had used a humidifier or any other inhaled aromatherapy from Day 1. Any volunteers who had received treatment with sleeping pills, sedatives, tranquilizers, muscle relaxants, opioids, or antidepressants in the 7 days prior to Day 1, with the exception of chronic medications taken at a stable dose for at least 3 months or longer, were excluded as were those who had received systemic corticosteroids or antibiotics within the 4 weeks prior to Day 1.

#### Treatment

Volunteers were randomized to take either two 600 mg ER guaifenesin or two matching placebo tablets every 12 hours with a full glass of water for seven consecutive days. The dose and duration were chosen to be consistent with the Mucinexproduct labeling, with 2400 mg being the maximum daily dose approved by the US Food and Drug Administration. Other OTC cough, cold, or allergy medicines were not permitted.

The first dose of study medication was taken at the clinic on Day 1 (following a 24-hour washout period if required). Subsequent doses were taken at home, except on Days 3 and 4 when patients took the morning dose at the investigational site. No specific instructions were given to the participants for the timing of the doses with respect to meals.

#### Assessments

PROs of efficacy, self-completed by patients, included the DCPD (Days 1–8 [or end of study]), the SSSA (Days 1, 3, 4, 8 [or end of study]) and the Wisconsin Upper Respiratory Symptom Survey (WURSS-21) (Days 1, 3, 4, 8 [or end of study]).

For the DCPD, participants rated changes in symptoms daily during the study including phlegm, cough, and lifestyle effects by answering 11 questions with one of five possible answers. Seven questions had possible responses of “never”, “rarely”, “sometimes”, “often”, or “always”; remaining questions had possible responses of “not at all”, “a little”, “somewhat”, “quite a bit”, or “extremely”.

For the SSSA, participants rated the severity of chest congestion, mucus thickness, and coughing on a scale of 0 to 5, with 0 equal to “none” and 5 equal to “as bad as it can be.”

The WURSS-21 was used to assess disease-specific changes in quality of life parameters during the study. This tool was included as it is a validated PRO for the assessment of quality of life changes in colds. The WURSS-21 includes one global severity item, 10 symptom-based items, nine functional items, and one global change item. The questionnaire was completed at baseline (before treatment), pre dose on Days 3 and 4 and at the Day 8 end of study visit.

On Day 8 of the study, participants completed the Patient’s End-of-Treatment Assessment. Participants provided an overall rating of the efficacy of the treatment in relieving symptoms associated with their infection by responding to the question: "Was the study medication effective?", Possible responses were: 0 = not effective at all, 1 = somewhat effective, 2 = moderately effective, 3 = very effective, 4 = extremely effective.

Also on Day 8, investigators completed the Investigator’s (Healthcare Professional; HCP) End-of-Study Assessment by responding “yes” or “no” to the question: “Based on the observed treatment outcomes for this patient, would you recommend the study medication for future use for the treatment of symptoms associated with an acute respiratory infection in this type of patient?”.

Objective measures of efficacy including sputum rheology, interfacial tension, and sputum volume were also assessed (not reported here). Safety assessments were performed by the investigator or investigational site personnel and consisted of assessment of vital signs and oral temperature and collection of adverse event (AE) information.

#### Statistical methods

All efficacy endpoints were exploratory and given equal consideration. The study sought to enroll 375 participants to ensure that 300 completed the study. Efficacy assessments were carried out on both the modified intent-to-treat (mITT) and the per protocol (PP) populations. Data for the mITT population are reported here.

The mITT population included all participants randomly assigned to treatment who received at least one dose of study medication and who had at least one efficacy assessment after baseline; it included all participants even those who had taken disallowed medications. In the mITT analysis, discontinued participants and those with missing data were included using the last observation carried forward approach. All safety analyses were conducted on the safety population, which included all enrolled participants who received at least one dose of study medication (active or placebo).

Responses to DCPD questions at each post-baseline assessment were compared between treatment groups using ordinal logistic regression with terms in the model for treatment group, study center and baseline. Within-patient changes from baseline to each post baseline assessment were assessed within each treatment group using the Wilcoxon signed rank test. In addition, to search for more sensitive study endpoints and population characteristics for possible follow-up studies, several post hoc exploratory analyses were conducted on composite endpoints defined as sums over subsets of the DCPD, and on selected subsets of the mITT population. These analyses compared the treatment groups using analysis of covariance (ANCOVA) models for changes from baseline, with terms in the model for treatment group, study center, and baseline.

Responses to SSSA questions at each post-baseline assessment were compared between treatment groups using ordinal logistic regression with terms in the model for treatment group, study center, and baseline. Within-patient changes from baseline (pre-dose on Day 1) to pre-dose on Days 3 and 4 and to Day 8, and from pre-dose on Days 3 and 4 to 3 hours after dosing on the same days were assessed within each treatment group using the Wilcoxon signed rank test, and compared between treatment groups using the Wilcoxon rank sum test.

The changes from baseline in each of the WURSS-21 assessments at each post-baseline day were compared between treatment groups using an ANCOVA with terms in the model for treatment group, study center, and baseline. Within-patient changes from baseline to each post-baseline assessment were assessed within each treatment group using a paired *t* test.

Responses to the Patient’s End-of-Treatment Assessment and the Investigator’s End-of-Study Assessment were compared between treatment groups using ordinal logistic regression with terms in the model for treatment group and study center. All statistical analyses were conducted using SAS version 8.2 or higher.

Because of the exploratory nature of the study, no multiplicity adjustments were made. Further, this study was not sized or intended to achieve statistical significant results across the board.

### Validation study

#### Study design and objectives

The validation study consisted of two phases: a content validity evaluation (Phase I) and a psychometric evaluation (Phase II). Phase I was a cross-sectional qualitative study in which each subject participated in a one-to-one qualitative interview, lasting approximately 1 hour. The objective of Phase I was to gather qualitative evidence to support the content validity of the DCPD (including the diary symptom subscale), the SSSA questions, and the Patient’s End-of-Treatment Assessment (results to be reported elsewhere).

Ethical approval was obtained from the IRB prior to the initiation of any study procedures. The study was conducted in accordance to the Declaration of Helsinki and all applicable federal, state, and local laws or regulations. All volunteers were required to provide written informed consent before participating in the study.

#### Subject selection (Phase I)

Volunteers were recruited through local websites and social networking sites from both the UK and the USA and analysis was performed at United BioSource Corporation (UBC), Bethesda, MD, USA. Participants were screened for study eligibility over the telephone (by trained UBC staff) against the inclusion and exclusion criteria described below.

Volunteers were included in Phase I if they had an URTI within 4 weeks prior to the study commencing with two or more of the following symptoms: cough, chesty cough, productive cough, excess mucus, difficulty coughing up mucus, thickened mucus, chest congestion, or any other description of a symptom relating to mucus, phlegm, or cough. Volunteers were required to read and understand English in order to review the PRO measures and respond to the interview questions.

Volunteers were excluded from the study if their respiratory symptoms were due to allergic rhinitis, chronic sinusitis, or chronic bronchitis (including acute exacerbations) and/or asthma or wheezing 4 weeks prior to the study commencing. Volunteers were also excluded if they had used antihistamines, intranasal medications, or inhaled corticosteroids in the past 4 weeks or had a history or diagnosis of chronic lung diseases including chronic obstructive pulmonary disease, bronchiectasis, or emphysema. Volunteers with any presence of cognitive impairment that would interfere with participating in a one-to-one interview (based on the screener’s opinion) were also excluded.

#### Validation content validity assessments (Phase I)

Each participant completed a single interview session either on the phone or in-person (Bethesda, MD, USA or London, UK) which was audio-recorded with participant consent. Phone interviews were conducted by trained UBC staff. During the first part of the interview, participants were asked open-ended questions about their symptom experiences with their recent URTI. This also allowed the interviewer to confirm cough and phlegm symptom experiences among participants with recent URTIs.

During the second part of the interview, participants completed the PRO instruments and were then engaged in a retrospective cognitive interview on these measures. These instruments included the DCPD, the SSSA questions and the Patient’s End-of-Treatment Assessment. The cognitive interview portion was designed to evaluate clarity of the items within the instruments; how the participants interpreted the items; assess the ease of completion of the instruments and test the appropriateness of the format, response scales and recall period used in the instruments.

#### Statistical analysis

All data collected were treated confidentially and all recorded interviews were transcribed and cross-checked. All quantitative data were double data entered and cleaned. A content analysis approach was used to analyze qualitative data from the interviews using ATLAS.ti analysis software (ATLAS.ti; version 5.0).

#### Validation psychometric evaluation (Phase II)

The objective of Phase II was to examine the psychometric properties of the DCPD, the SSSA items (cough, congestion, mucus), and the Patient’s End-of-Treatment Assessment. Descriptive and instrument score characteristics, reliability, validity, and ability to detect change, and score interpretation of the PROs, consistent with current regulatory standards [10,11], were evaluated.

Phase II psychometric evaluations used blinded data, pooled across treatment groups, from the pilot clinical study (above) assessments conducted at Days 1, 4 and Day 8 or end of study. Statistical analyses were conducted in accordance with classical psychometric theory [12]. Internal consistency reliability was evaluated with Cronbach’s alpha statistic [13]. Convergent validity was evaluated by examining the magnitude of correlations between the DCPD and conceptually related measures at the same assessment point.

## Results

### Clinical pilot study

#### Patient population

**Figure 1:**
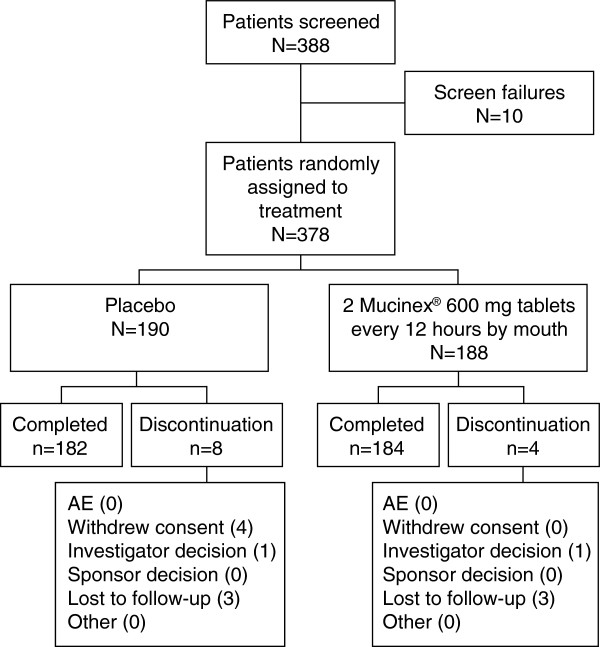
Patient disposition (all-patient population).

A total of 378 participants with a mean age of 41.0 ± 14.47 years (range 13.0 − 85.0 years) were randomly assigned to treatment (all-patient population) and 366 participants completed the study (Figure 1). There were 369 patients in the mITT population and 377 patients in the safety population. The treatments were well balanced with respect to demographics and baseline characteristics (Table 1).

**Table 1:** Patient demographics and baseline characteristics

	**Placebo**	**ER guaifenesin**	**Overall**
	**(n = 190)**	**(n = 188)**	**(N = 378)**
**Mean age, years ± SD**	40.8 ± 15.04	41.1 ± 13.91	41.0 ± 14.47
**Age range, years**	13 – 83	18 – 85	13 – 85
**Sex, male %**	55.8	48.9	52.4
**Sex, female %**	44.2	51.1	47.6
**Race, White %**	61.1	60.6	60.8
**Black %**	33.2	36.7	34.9
**Asian %**	4.2	1.6	2.9
**American Indian or Alaska Native %**	1.6	0.5	1.1
**Other %**	0	0.5	0.3
**Ethnicity, Hispanic or Latino %**	5.3	7.4	6.3
**Not Hispanic or Latino %**	94.7	92.6	93.7
**Onset day of cold symptoms before study entry**			
**−8 days %**	0.5	0	0.3
**−6 days %**	0.5	0	0.3
**−5 days %**	3.7	5.3	4.5
**−4 days %**	26.8	19.7	23.3
**−3 days %**	39.5	38.3	38.9
**−2 days %**	21.1	32.4	26.7
**−1 days %**	7.4	4.3	5.8
**0 days %**	0.5	0	0.3
**Onset day of productive cough before study entry**			
**−7 days %**	0.5	0	0.3
**−5 days %**	0	0.5	0.3
**−4 days %**	2.6	1.1	1.9
**−3 days %**	14.7	10.1	12.4
**−2 days %**	46.3	50.5	48.4
**−1 days %**	31.1	34.0	32.5
**0 days %**	4.7	3.7	4.2
**Diagnosis before study entry**			
**Acute nasopharyngitis/rhinopharyngitis %**	81.6	83.0	82.3
**Acute bronchitis %**	10.0	6.9	8.5
**Acute sinusitis %**	4.7	6.9	5.8
**Acute pharyngitis %**	2.6	2.1	2.4
**Acute laryngitis %**	0.5	0.5	0.5
**Other %**	0.5	0.5	0.5

*SD*: standard deviation. Note: Onset day is relative to first dose date and all percentages are based on the number of patients in each treatment group.

#### Efficacy evaluation

The sample size was selected to look for indications and trends without the expectation of seeing p-values of < 0.05. Any achievement of these values was considered a very strong signal.

In general, subjective measures of efficacy at Day 4 showed the most prominent difference between treatment groups. Analysis of total scores showed no significant differences. However, analysis of individual questions from the DCPD and SSSA showed several statistically significant differences between treatment groups for questions relating to cough, in favor of ER guaifenesin in the mITT population (Tables 2 and 3). Significant differences also noted on Days 5 and 7 (Table 2). Although the main cluster of efficacy signals was around Days 4/5, there were also signs of an early onset of effect at earlier time points.

**Table 2:** Summary of DCPD between-treatment comparisons (mITT population)

	**Study Day**						
** Questions**	**2**	**3**	**4**	**5**	**6**	**7**	**8**
**1. Over the last 24 hours, how often did you bring up phlegm?**							
**2. Over the last 24 hours, how often did your phlegm make it difficult for you to breathe?**				p < 0.10			
**3. Over the last 24 hours, how often did you feel uncomfortable about bothering other people while bringing up phlegm?**							
**4. Over the last 24 hours, how annoyed were you by your phlegm?**							
**5. Over the last 24 hours, how often did your phlegm interfere with your ability to speak?**			p < 0.05				
**6. Over the last 24 hours, how often did your phlegm prevent you from going to public places?**							
**7. Over the last 24 hours, how often did you have to interrupt your usual activities to get rid of your phlegm?**							
**8. Over the last 24 hours, how thick was your phlegm?**			p < 0.05				
**9. Over the last 24 hours, how difficult was it for you to bring up phlegm?**				p < 0.10			
**10. How much did you cough when you woke up in the morning?**		p < 0.10	p < 0.05	p < 0.05		p < 0.10	
**11. How often did you cough during the day today?**			p < 0.05			p < 0.05	p < 0.10

*DCPD*: Daily Cough and Phlegm Diary; *mITT*: modified intent-to-treat. p < 0.05: statistically significant difference between treatment groups; p < 0.10: viewed as a trend towards a significant difference between treatment groups. Note: p-values are from an ordinal logistic regression model with predictors: treatment group, center, and baseline.

**Table 3:** Summary of SSSA between-treatment comparisons (mITT population)

	**Day 3**	**Day 3**	**Day 4**	**Day 4**	**Day 8**
	**Hour 0**	**Hour 3**	**Hour 0**	**Hour 3**	
**Congestion**					
**Mucus**	p < 0.10		p < 0.10		p < 0.10
**Cough**			p < 0.05		

*SSSA*: Spontaneous Symptom Severity Assessment; *mITT*: modified intent-to-treat. p < 0.05: statistically significant difference between treatment groups; p < 0.10: viewed as a trend towards a significant difference between treatment groups. Note: p-values are from Wilcoxon rank sum tests comparing treatments. Results for Hour 0 on Days 3 and 4 and for Day 8 are treatment comparisons based on within-subject changes from Baseline (Day 1). Results for Hour 3 on Days 3 and 4 are treatment comparisons based on within-subject changes from Hour 0 of the same day.

The DCPD assessment of symptoms also indicated advantages for ER guaifenesin over placebo for the between-day changes from baseline in response to the questions “Over the last 24 hours how often did your phlegm prevent you from going to public places?” (Day 2; p = 0.0016) and “Over the last 24 hours, how difficult was it for you to bring up phlegm?” (Day 5; p = 0.0070).

As expected with this acute indication, over time, the DCPD improved for all patients, regardless of treatment group; similarly the SSSA showed that the severity of chest congestion, mucus thickness, and cough improved for all patients over time, regardless of treatment group. For WURSS-21 quality of life assessments, the total score decreased at a similar rate over time in both treatment groups and at each visit, the scores between the treatment groups were similar, with no statistical differences between the groups at any assessment.

**Figure 2:**
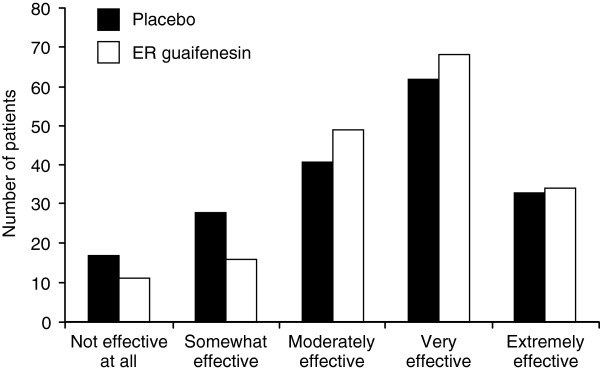
Patient’s End-of-Treatment Assessment (mITT population) for the question “Was the study medication effective?”.

The Investigator’s (HCP) End-of-Study Assessment showed that, based on the observed treatment outcome, 91.6% would recommend ER guaifenesin and 82.8% would recommend placebo (mITT; p = 0.0096) for future use for the treatment of symptoms associated with acute URTI in this type of patient. For the Patient’s End-of-Treatment Assessment, results somewhat favored ER guaifenesin but the comparison with placebo was not statistically significant (p = 0.1712). The placebo effect was high, with 73.0% of patients finding placebo at least moderately effective (Figure 2).

Exploratory analyses were performed on the DCPD data to evaluate the relative sensitivity of selected composite endpoints to discriminate between treatments for future studies; three different subsets were examined (Table 4). The 8-symptom related questions in the DCPD, analyzed as a composite score (questions 1, 2, 4, 5, 8, 9, 10 and 11 from the 11 questions) appeared to be the strongest candidate endpoint for further evaluation (SUM8). The SUM8 composite seemed to be better suited to capture the early onset of effect and show statistically and clinically meaningful differences compared with placebo. These results suggested that a cumulative measure (e.g., a repeated measures algorithm), may be the best approach to capturing the full impact of an expectorant drug on cough and phlegm symptoms.

**Table 4:** Summary of ad hoc analyses for DCPD total score, mean change from baseline at Day 4

** Endpoint**	**Placebo**	**ER guaifenesin**	**p-value**
**1. Excluding questions 3, 6, and 7 (SUM8)**			
Overall	**−5.7**	**−7.1**	**0.0372**
1 day since onset of symptoms	**−6.2**	**−8.4**	**0.0339**
2 days since onset of symptoms	−6.1	−7.5	0.2281
3 days since onset of symptoms	−5.6	−6.2	0.4424
4 days since onset of symptoms	−5.4	−7.5	0.2475
5 days since onset of symptoms	−7.9	−8.0	0.6814
Diagnosis: acute rhinopharyngitis	−5.9	−6.8	0.1521
Diagnosis: acute sinusitis	−3.0	−8.0	0.0534
Diagnosis: acute pharyngitis	−15.3	−5.5	NA
Diagnosis: acute bronchitis	**−4.3**	**−9.8**	**0.0064**
**2. Including questions 5, 8, 10, and 11 only**			
Overall	**−3.0**	**−3.9**	**0.0059**
1 day since onset of symptoms	**−2.9**	**−4.6**	**0.0093**
2 days since onset of symptoms	−3.5	−4.2	0.1499
3 days since onset of symptoms	−2.7	−3.4	0.1439
4 days since onset of symptoms	−2.8	−4.0	0.1342
5 days since onset of symptoms	−5.3	−4.3	0.5617
Diagnosis: acute rhinopharyngitis	**−3.0**	**−3.8**	**0.0263**
Diagnosis: acute sinusitis	−1.8	−4.3	0.0715
Diagnosis: acute pharyngitis	−8.7	−3.5	NA
Diagnosis: acute bronchitis	**−2.1**	**−4.7**	**0.0368**
**3. Including questions 8, 9, 10, and 11 only**			
Overall	**−2.5**	**−3.2**	**0.0062**
1 day since onset of symptoms	**−1.8**	**−4.4**	**0.0286**
2 days since onset of symptoms	−2.6	−3.4	0.0965
3 days since onset of symptoms	−2.7	−2.6	0.4421
4 days since onset of symptoms	−2.4	−3.1	0.2021
5 days since onset of symptoms	−3.4	−4.6	0.6586
Diagnosis: acute rhinopharyngitis	**−2.5**	**−3.2**	**0.0176**
Diagnosis: acute sinusitis	−0.6	−2.6	0.1035
Diagnosis: acute pharyngitis	−8.0	−2.3	NA
Diagnosis: acute bronchitis	**−2.1**	**−4.0**	**0.0330**

*DCPD*: Daily Cough and Phlegm Diary. Note: Items with statistically significant values are in bold. P-value is from analysis of covariance (ANCOVA) model comparing treatments within day with predictors of treatment group, center, and baseline. NA indicates that the p-value in not estimable.

This 8-question composite is likely to have better statistical distribution properties than the smaller composites of 4 questions that were evaluated or each question alone. The composite questions evaluated conceptually related but different concepts, and the questions were not redundant (e.g., measuring the same concepts), thereby adding unique information to the scale.

#### Safety

The mean duration of exposure was 7.0 (range 6 − 9) days in the ER guaifenesin group and 7.1 (range 0 − 18) days in the placebo group. The treatment was well tolerated and treatment-emergent AEs were reported in only 8.5% of patients in the ER guaifenesin group and 5.3% of patients in the placebo group (Table 5). Most events were mild in severity and resolved without intervention. None of the AEs were deemed definitely related to study medication and none of the study discontinuations were deemed to be due to AEs.

**Table 5:** Summary of treatment-related (possible/probable) AEs (all causalities), by system organ class and preferred term, occurring in ≥ 0.5% of patients in either treatment group (safety population)

	**Placebo**		**ER guaifenesin**	
** System organ class**	**(n = 189)**		**(n = 188)**	
** Preferred term**	**Possible**	**Probable**	**Possible**	**Probable**
**Total number of treatment-emergent AEs**	4	2	7	5
**Number of unique patients with at least one AE**	2.1	1.1	3.7	2.7
**Gastrointestinal disorders %**	1.6	0.5	0.5	1.6
**Abdominal discomfort %**	0.5	0	0	0
**Diarrhoea %**	0.5	0	0	0
**Dry mouth %**	0	0.5	0	0
**Nausea %**	0	0	0.5	1.6
**Vomiting %**	0.5	0	0	0
**Nervous system disorders %**	0	0.5	3.2	0.5
**Headache %**	0	0.5	2.1	0.5
**Sinus headache %**	0	0	0.5	0
**Somnolence, %**	0	0	0.5	0
**Psychiatric disorders %**	0	0	0	0.5
**Insomnia %**	0	0	0	0.5
**Skin and subcutaneous tissue disorders %**	0.5	0	0	0
**Rash %**	0.5	0	0	0

*AE*: adverse event. Note: The total number of AEs includes all treatment-emergent AEs for patients. Patients may have more than one treatment-emergent AE per system organ class and preferred term. At each level of patient summarization, a patient was counted once for the most related AE.

### Validation study

A total of 12 participants were interviewed during Phase I. The mean age of the participants was 34.3 ± 9.7 years (range 24.0 – 50.0 years). Half of the participants were male and 58% were white, 25% black and 17% Asian. None of the participants reported having any existing health conditions and 92% were not on any medications (one participant reported using NuvaRing [Merck and Co, Inc., USA] birth control).

The results from the interviews in Phase I generally supported the content of the DCPD, the SSSA, and the Patient’s End-of-Treatment Assessment. The open-ended questions revealed important symptoms of URTIs including cough (productive and non-productive), sputum, and chest congestion. All of the more specific items evaluated in the DCPD, such as difficulty bringing up phlegm and thickness of phlegm, were discussed as important symptoms associated with URTI.

Diary items were generally well understood by the participants, were easy for participants to respond to and were highly relevant. There were, however, some minor inconsistencies in participants’ interpretation of terminology; for example, phlegm was considered to be an ambiguous term. Similar results were found for the SSSA items and the Patient’s End-of-Treatment Assessment items, which were well understood but required some further explanation around selected terms.

A total of 322 patients with baseline data were included in the analysis during Phase II. The mean age of the participants was 41.9 ± 14.4 years (range 13.0 – 85.0 years). Sex was approximately equally split (males, 53.1%; females, 46.9% and the majority of patients were white (55.9%) or black (39.1%) along with 3.4% Asian patients.

Data were excluded from one site which had “questionable practices” (n = 19) and from some patients who were provided the wrong questionnaire (n = 37).

Cronbach’s alpha estimates for the SUM8 score were excellent, with estimates exceeding 0.70 at every time point (Table 6). Validity estimates demonstrated good convergent, discriminant, and known group’s validity (Table 7).

**Table 6:** Internal consistency reliability symptom subscale (items 1, 2, 4, 5, 8, 9, 10 and 11) score at Days 1, 4, and 8 (Cronbach’s Alpha)

** Item**	**Cronbach’s alpha**			**Cronbach’s alpha with item deleted**		
	**Day 1**	**Day 4**	**Day 8**	**Day 1**	**Day 4**	**Day 8**
Subscale score alpha	0.76	0.87	0.90			
	(n = 310)	(n = 305)	(n = 290)			
1. Bring up phlegm				0.73	0.84	0.88
2. Difficult to breathe				0.72	0.84	0.88
4. Annoyed by phlegm				0.71	0.83	0.87
5. Interference with ability to speak				0.71	0.84	0.88
8. Phlegm thickness				0.72	0.85	0.88
9. Difficulty bringing up phlegm				0.81	0.90	0.93
10. Cough when woke up				0.73	0.84	0.88
11. Cough during day				0.74	0.84	0.88

**Table 7:** Convergent validity: correlations between more and less conceptually related measures at Days 1, 4 and 8

	**Daily Cough and Phlegm Diary – 8 items**		
	**Symptom subscale – Day 1**	**Symptom subscale – Day 4**	**Symptom subscale – Day 8**
Spontaneous symptom assessment: congestion	0.15*	0.50‡	0.64‡
Spontaneous symptom assessment: mucus	0.19†	0.42‡	0.59‡
Spontaneous symptom assessment: cough	0.20†	0.52‡	0.57‡
Patient’s End-of-Treatment Assessment	0.07	−0.17*	−0.32‡
WURSS-21 Total	0.50‡	0.68‡	0.70‡
WURSS-21 Symptom	0.44‡	0.65‡	0.69‡
WURSS-21 Function	0.45‡	0.61‡	0.61‡
Investigator’s End-of-Study Assessment	−0.04	−0.20†	−0.37‡

*WURSS-21*: the Wisconsin Upper Respiratory Symptom Survey. Spearman rank-order correlations: *p < 0.01, †p < 0.001, ‡p < 0.0001.

## Discussion

Expectorants are an important and widely used component of treatment for relief of the symptoms of acute URTIs. Currently the main assessments used to assess mucoactive therapies include quality of life questionnaires [14,15]; however, there is a need for a universally accepted, sensitive, clinically relevant and appropriately validated PRO measure for use as the primary efficacy outcome measure in future clinical trials in this field.

The clinical pilot study reported here is unique in the field of guaifenesin research and was conducted to assess the efficacy and safety of ER guaifenesin compared with placebo in patients with productive cough due to an acute URTI. In addition, together with the validation study, the research was conducted to support the selection of primary endpoints for future clinical studies of mucoactive compounds.

The subjective measures of efficacy in this study showed the most prominent difference between groups at Day 4 but there were also some improvements at earlier time points. In agreement with the mode of action of ER guaifenesin and current labeling in the USA, the most significant symptom improvements were seen with cough and several discomforts associated with excess and tenacious mucus.

The rating of effects on mucus in the SSSA showed a strong trend throughout the course of treatment but did not quite meet statistical significance at the p < 0.05 level. Nevertheless, the consistency of the signal over the course of treatment supports the concept of a steady effect of ER guaifenesin on airway mucus; this is the mechanism by which guaifenesin improves mucus-related symptoms as reported in questions 2, 5 and 8 of the DCPD.

The DCPD includes 8-symptom related questions (SUM8) and three functional items that focus on phlegm and cough. Due to the fact that excess and tenacious airway mucus during URTIs is one of the triggers for cough, it also seems to be consistent that ER guaifenesin was associated with improvements in cough symptoms on Day 4 (p = 0.0293). This signal weakened and lost statistical significance towards Day 8 (p = 0.1158), which can be explained by the overall improvement of other cough triggers and disease dynamics of URTIs over time.

In this study the WURSS-21 score decreased at a similar rate and showed statistically significant between-day comparisons for both treatment groups. However, the WURSS-21 is a quality of life questionnaire designed to assess the negative impact of the many symptoms of the common cold [16] rather than the symptomatic effects specific to mucus; therefore, it does not highlight the specific improvements associated with expectorant treatment. This suggests that this tool may not be suitable for future studies of guaifenesin.

For the Patient’s End-of-Treatment Assessment of outcomes there was no statistically significant difference between treatment groups; however, it did show a trend favoring ER guaifenesin and the difference between treatments (9.6%) comes close to a meaningful therapeutic effect. The lack of a statistically significant difference between the treatment groups may have been due to a relatively small sample size and large placebo effect.

The Investigator’s (HCP) End-of-Study Assessment significantly favored ER guaifenesin over placebo; although this outcome is not likely to be an appropriate primary measure of treatment efficacy it may be helpful in confirming some of the improvement in symptoms and could be considered as a secondary endpoint in future studies.

The clinical pilot study confirmed that treatment with ER guaifenesin was well tolerated in accordance with the well-documented safety profile and post-marketing surveillance of an over-the-counter ER, bilayer formulation of guaifenesin.

Based on the overall results from the pilot study, the most promising tools for discriminating symptomatic improvements between the active treatment and placebo were found to be symptom self-assessments by the patient, i.e. the DCPD and the SSSA with peak separation at Days 4 and 5. Additional post hoc analyses of the data indicated that the best way to discriminate between the active and placebo treatments is to use a composite sub-score of the questions in the DCPD; the ‘SUM8’, limited to the symptoms questions numbered 1, 2, 4, 5, 8, 9, 10, and 11, seemed best suited to capture the effects of the treatment.

Results from the validation study provided further evidence that SUM8 is likely to be a sensitive and precise measure for evaluating changes in URTI respiratory symptoms over time with an expectorant treatment, demonstrated by the reliability and validity estimates. The SUM8 scale demonstrated sensitivity to detect changes over time with patient ratings of efficacy. During interviews some minor inconsistencies in the interpretation of the terminology were found, therefore, future studies could provide patients with training and/or a glossary that further defines any potentially ambiguous terms.

It was determined that the SUM8 is a comprehensive symptom measure that evaluates aspects of phlegm experience, with six items dedicated to this concept. As ER guaifenesin is an expectorant that improves the rheology and clearance of respiratory tract mucus, this comprehensive measure of phlegm may be optimal for precisely evaluating treatment effects.

Results suggest that from the patient’s perspective, a clinically meaningful change in the SUM8 might be approximately 4.58 points. This score represents an intra-individual Minimally Important Difference (MID). A limitation of this analysis was that, due to the population being studied and the natural disease progression of URTIs, study design, and available measures, it was not possible to adequately evaluate test–retest reliability estimates.

## Conclusions

In summary, the validation study showed that the SUM8 diary scores were robust and suitably reliable for use as efficacy endpoints in studies of expectorants such as guaifenesin for the symptoms of URTIs. Given the mechanism of action of ER guaifenesin and robust psychometric results for the SUM8, it is recommended that the 8-symptom related questions be prioritized for future efficacy studies.

Further improvements in the power of future studies will come from more targeted sample size estimates and further refinement of the inclusion/exclusion criteria. Future clinical studies with guaifenesin will confirm whether these are viable assessment tools and suitable primary outcome measures for the evaluation of the treatment of patients with URTIs.

## Abbreviations

AE: Adverse event; ANCOVA: Analysis of covariance; DCPD: Daily cough and phlegm diary; ER: Extended release; HCP: Healthcare professional; MID: Minimally important difference; mITT: Modified intent-to-treat; PP: Per protocol; PRO: Patient-reported outcome; SSSA: Spontaneous Symptom Severity Assessment; SUM8: 8-symptom related questions; URTI: Upper respiratory tract infection; WURSS-21: Wisconsin Upper Respiratory Symptom Survey.

## Competing interests

HA is a consultant to Reckitt Benckiser, MV is an employee of United BioSource Corporation, a company that receives payment from Reckitt Benckiser for services provided and GS is an employee of Reckitt Benckiser.

## Authors’ contribution

HA contributed to the pilot study design, interpretation of results and writing/reviewing of the final study report. MV contributed to the validation study design, statistical analysis, interpretation of results and writing of the study report. GS contributed to the pilot study design, conduct and reporting. All authors read and approved the final manuscript.
